# Assessment of Differences in Inpatient Rehabilitation Services for Length of Stay and Health Outcomes Between US Medicare Advantage and Traditional Medicare Beneficiaries

**DOI:** 10.1001/jamanetworkopen.2020.1204

**Published:** 2020-03-18

**Authors:** Ying Cao, Jing Nie, Sue Ann Sisto, Paulette Niewczyk, Katia Noyes

**Affiliations:** 1 Division of Health Services Policy and Practice, Department of Epidemiology and Environmental Health, University at Buffalo, Buffalo, New York; 2 Department of Rehabilitation Science, University at Buffalo, Buffalo, New York; 3 Uniform Data System for Medical Rehabilitation, University at Buffalo, Buffalo, New York

## Abstract

**Importance:**

Enrollment in Medicare Advantage (MA) has been increasing and has reached one-third of total Medicare enrollment. Because of data limitations, direct comparison of inpatient rehabilitation services between MA and traditional Medicare (TM) beneficiaries has been very scarce. Subgroups of elderly individuals admitted to inpatient rehabilitation facilities (IRFs) may experience different care outcomes by insurance types.

**Objective:**

To measure the differences in length of stay and health outcomes of inpatient rehabilitation services between TM and MA beneficiaries in the US.

**Design, Setting, and Participants:**

This multiyear cross-sectional study used the Uniform Data System for Medical Rehabilitation to assess rehabilitation services received by elderly (aged >65 years) Medicare beneficiaries in IRFs between 2007 and 2016 for stroke, hip fracture, and joint replacement. Generalized linear models were used to assess whether an association existed between Medicare insurance type and IRF care outcomes. Models were adjusted for demographic characteristics, clinical conditions, and facility characteristics. Data were analyzed from September 2018 to August 2019.

**Exposures:**

Medicare insurance plan type, TM or MA.

**Main Outcomes and Measures:**

Inpatient length of stay in IRFs, functional improvements, and possibility of returning to the community after discharge.

**Results:**

The sample included a total of 1 028 470 patients (634 619 women [61.7%]; mean [SD] age, 78.23 [7.26] years): 473 017 patients admitted for stroke, 323 029 patients admitted for hip fracture, and 232 424 patients admitted for joint replacement. Individuals enrolled in MA plans were younger than TM beneficiaries (mean [SD] age, 76.96 [7.02] vs 77.95 [7.26] years for stroke, 79.92 [6.93] vs 80.85 [6.87] years for hip fracture, and 74.79 [6.58] vs 75.88 [6.80] years for joint replacement) and were more likely to be black (17 086 [25.5%] vs 54 648 [17.9%] beneficiaries) or Hispanic (14 496 [28.5%] vs 24 377 [8.3%] beneficiaries). The MA beneficiaries accounted for 21.8% (103 204 of 473 017) of admissions for stroke, 11.5% (37 160 of 323 029) of admissions for hip fracture, and 11.8% (27 314 of 232 424) of admissions for joint replacement. The MA beneficiaries had shorter mean lengths of stay than did TM beneficiaries for both stroke (0.11 day; 95% CI, −0.15 to −0.07 day; 1.15% shorter) and hip fracture (0.17 day; 95% CI, −0.21 to −0.13 day; 0.85% shorter). The MA beneficiaries also had higher possibilities of returning to the community than did TM beneficiaries, by 3.0% (95% CI, 2.6%-3.4%) for stroke and 5.0% (95% CI, 4.4%-5.6%) for hip fracture. The shorter length of stay and better ultimate outcomes were achieved without substantially compromising the intermediate functional improvements. Facility type (freestanding vs within an acute care hospital) and patient alternative payment sources other than Medicare (none vs other) partially explained the differences between insurance types.

**Conclusions and Relevance:**

This study suggests that MA enrollees experience shorter length of stay and better outcomes for postacute care than do TM beneficiaries in IRFs. The magnitude of the differences depends on treatment deferability, patient sociodemographic subgroups, and facility characteristics.

## Key Points

**Question:**

Do Medicare Advantage beneficiaries experience different care services and outcomes from inpatient rehabilitation facilities than traditional Medicare beneficiaries do?

**Findings:**

In this multiyear cross-sectional study of more than 1 million inpatient rehabilitation facility admissions (473 017 for stroke, 323 029 for hip fracture, and 232 424 for joint replacement), Medicare Advantage beneficiaries had a shorter mean length of stay (1.15% shorter for stroke and 0.85% shorter for hip fracture) and a greater likelihood of returning to the community (3.0% for stroke and 5.0% for hip fracture) than did traditional Medicare beneficiaries, without substantially compromising their functional improvements.

**Meaning:**

These findings suggest that policy makers may consider using strategies in managed care to further improve care quality and control costs.

## Teaser

This cross-sectional study measures the differences in length of stay at inpatient rehabilitation services and health outcomes between traditional Medicare and Medicare Advantage beneficiaries.

## Introduction

In the US, approximately 42% of all hospitalized Medicare beneficiaries receive postacute care (PAC) after discharge; among those, 5.5% go to inpatient rehabilitation facilities (IRFs).^1^ In 2015, US Medicare spending on PAC for traditional Medicare (TM) beneficiaries was $60.3 billion, accounting for approximately 10% of total national health care spending, of which $7.5 billion was spent in IRFs, representing 13% of the total PAC spending.^2^ In addition to IRFs, PAC can also be provided by skilled nursing facilities (SNFs), home health agencies, and long-term care hospitals. Compared with other PAC sectors, such as SNFs and home health agencies, which experienced a more pronounced decline in annual spending growth than total Medicare spending (−2.8% and −1.8% vs 0.6%), the mean annual spending growth for IRFs was fairly stable at a higher rate (1.8%) between 2008 and 2015.^1,3^

Medicare Advantage (MA), the managed care version of Medicare, controls care costs via management of network (ie, selective contracting with practitioners), utilization (eg, preauthorization), and incentives (eg, capitated payments). The percentage of Medicare beneficiaries enrolled in MA plans has almost doubled over the past decade, from approximately 16% in 2006 to 31% in 2015, and continues to grow.^4,5^

As a result of limitations on data availability, direct comparisons between TM^6^ and MA in terms of PAC delivery and outcomes are very scarce, with only a few exceptions, such as the study by Huckfeldt et al^7^ on hospital discharge patterns to alternative PAC facilities, including IRFs, SNFs, and home health agencies. Even less is known about the differences in utilization, costs, and health outcomes between TM and MA. The present study seeks to answer these questions and to contribute to health services research on PAC in several ways.

First, we measured the differences between TM and MA with respect to inpatient rehabilitation services on care utilization^8,9^ and patient health outcomes.^10,11^ Second, we sought to fill the knowledge gaps by understanding functional outcomes among Medicare beneficiaries who receive inpatient rehabilitation services. Because of data limitations and different measurement requirements across reporting and administrative systems, rehabilitation sector–specific care outcomes (eg, functional status) have rarely been available for both TM and MA beneficiaries.^12,13^ Third, this study is also closely related to the general literature on the scientific inquiry of the mechanisms that contribute to the differences in care delivery and outcomes between TM and MA.^14,15,16^ This study attempted to disentangle and quantify the relative strength of patient-level, facility-level, and regional care system–level variations that contribute to the differences between TM and MA in inpatient rehabilitation services.

Understanding the differences between TM and MA in terms of PAC quality, cost, and outcomes is critical given the current growth of alternative payment models and accountable care organizations,^17^ as well as the recently proposed Medicare reforms that shift the traditional fee-for-service payment model to capitated plans with payment incentives similar to those in MA.^5,18,19,20^ Equally important is the identification of patient, facility, and regional characteristics associated with poor care quality and outcomes, which will inform future implementation, quality improvement, and policy reform.^21,22,23^

## Methods

### Data Sources

The Uniform Data System for Medical Rehabilitation (UDSMR) is the world’s largest nongovernment data repository for inpatient medical rehabilitation in the US since 1987.^24^ Data from the Inpatient Rehabilitation Patient Assessment Instrument, a tool for patient assessment used at both admission and at discharge mandated by the Centers for Medicare & Medicaid Services for payment reimbursement,^25^ was obtained from the UDSMR data repository. The Inpatient Rehabilitation Patient Assessment Instrument includes patient-level sociodemographic variables, prehospital living arrangement, marital status, predisability employment status, discharge disposition, diagnoses (*International Classification of Diseases, Ninth Revision* codes), facility characteristics, and cost factors such as length of stay (LOS) and source of payment. In addition, the Functional Independence Measure (FIM) instrument is also included in the Inpatient Rehabilitation Patient Assessment Instrument, which includes items on patient level of physical and cognitive functioning assessed by a clinician from an IRF.

### Study Sample

 The study was approved by the institutional review board of University at Buffalo, which also waived the need to obtain informed consent from the participants because the data were anonymous. This study follows the Strengthening the Reporting of Observational Studies in Epidemiology (STROBE) reporting guideline.

This study focused on elderly (aged >65 years) adult Medicare beneficiaries who received initial rehabilitation services (readmission and facility transfers excluded) between 2007 and 2016 from more than 1100 Medicare-reimbursable IRFs across the US. Three common reasons for IRF stays were selected in this study according to UDSMR Impairment Group Codes: 2 acute conditions (stroke, codes 01.1-9; and hip fracture, codes 08.11-12 and 08.4) and 1 elective condition (joint replacement, codes 08.51-52, 08.61-62, and 08.71-72), which accounted for approximately 19.8%, 11.5%, and 6.8% of all inpatient rehabilitation services delivered to the aging population, respectively.^2^

Comparing care delivery in IRFs between TM and MA beneficiaries faces the challenges of endogeneity and selection, because patients could self-select to enroll in TM or MA plans (eg, MA enrollees are, on average, healthier than TM enrollees); furthermore, MA plans could affect the probability that a patient is admitted to a hospital and then to an IRF. Such selection concerns were less of an issue for acute conditions than elective conditions. As a result, this study focuses on the results for the 2 acute conditions, and the results for the elective condition (joint replacement) are shown in eTable 1 and eTable 2 in the Supplement for reference.

The sample started with 1 199 229 patient records that passed the aforementioned inclusion criteria. We excluded patients whose prehospital living settings were nonhome (ie, institutionalized aging groups, 33 568 individuals), patients who died during the rehabilitation stays (1584 individuals), patients whose inpatient stay for rehabilitation was longer than 30 days (18 554 individuals) or shorter than 3 days (18 399 individuals), and those with key variables missing (eg, age and sex, 32 individuals). Beneficiaries with dual Medicare and Medicaid coverage (98 622 individuals) were also excluded because dual eligibility was not equally distributed between TM and MA beneficiaries, and the exclusion can mitigate the problem of unobserved heterogeneity. The Figure shows a flowchart of the sample construction.

**Figure.**
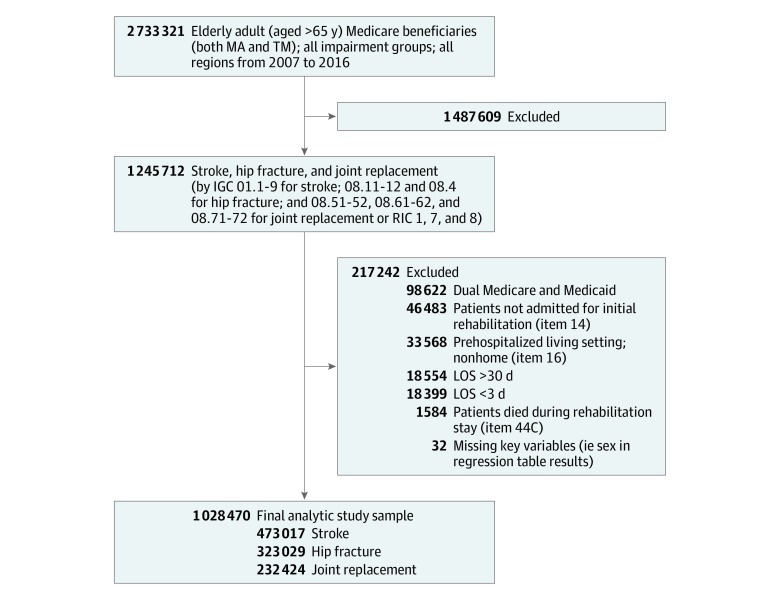
Study Sample Flowchart No patient had a length of stay (LOS) greater than 3 SDs above or below the logarithm of LOS after exclusion for greater than 30 days or less than 3 days. Item numbers refer to the Inpatient Rehabilitation Patient Assessment Instrument. IGC indicates Impairment Group Codes; MA, Medicare Advantage; RIC, Rehabilitation Impairment Category; and TM, traditional Medicare.

### Outcome Variables

#### Length of Stay

Rehabilitation LOS was calculated as the total number of days spent in the inpatient rehabilitation facility excluding the interrupted periods. The LOS was used as a proxy of resource utilization.^11,12^

#### Functional Status

A patient’s motor and cognitive function were assessed within 72 hours of admission and discharge using the FIM instrument. The FIM instrument consists of 18 items covering 6 domains: self-care or activities of daily living (6 items), bladder and bowel control (2 items), mobility (3 transfer items), locomotion (2 items on walking or wheelchair use and stairs), communication (2 items on comprehension and expression), and social cognition (3 items on social interaction, problem solving, and memory). The first 4 domains measure motor function and the last 2 domains measure cognitive function.

All 18 items are measured on a 7-point scale from 1 (total assistance) to 7 (complete independence). The motor function score ranges from 13 to 91 and the cognitive function score ranges from 5 to 35, with the total FIM score ranging from 8 to 126.^13,26^

The FIM score at admission indicated the severity of a patient’s acute condition before treatment, and its mean value of a subpopulation reflected the inpatient admission requirement. The FIM admission score was used as one of the clinical factor control variables in the analysis.

The change in FIM, which was obtained by subtracting a patient’s FIM at admission from the FIM at discharge, was used to assess the effectiveness of an inpatient stay. A larger FIM score improvement indicated higher treatment effectiveness. The total FIM score change divided by total LOS represented the mean daily efficiency of treatment.^26^

#### Discharge to Community

The discharge setting was collapsed into a dichotomized variable. The variable was return to the community (ie, home settings) as opposed to discharge to another level of PAC, readmission to acute care, or discharge to residence in an institutional setting.

### Control Variables

Control variables in the study included patient demographic characteristics, clinical factors, and facility characteristics. Demographic characteristics included age, sex, race/ethnicity, marital status, and secondary payer source (having other sources of payment vs none), year, and region of inpatient stay as fixed effects. Clinical factors included FIM score at admission, comorbidity tiers, and case-mix group defined by Centers for Medicare & Medicaid Services.^25^ Facility characteristics included number of certified beds and facility type (ie, whether an IRF is a stand-alone facility or a unit within the same acute care facility). These variables were selected according to the availability of the UDSMR data set and other studies on rehabilitation outcomes.^11,12,27,28^

### Statistical Analysis

The study adopted a retrospective study design to compile multiyear admissions to IRFs between 2007 and 2016. The unit of observation is 1 patient-episode. The main area of interest was whether and by how much the inpatient rehabilitation care delivery and outcomes were different between beneficiaries who enrolled in MA and TM after controlling for other patient-level, facility-level, and regional-level differences. The model specification is outcome = *I*(*MA*)∙θ + *X*β + *Z*δ + λ + τ + ϵ, where *I*(*MA*) is a dummy indicator (equal to 1 if the patient record was primarily paid by an MA plan, and 0 if it was paid primarily by TM fee-for-service), the estimated coefficient θ captures the impact of Medicare insurance type (MA vs TM) on outcomes of interest, *X*β represents patient-level demographic characteristics and clinical conditions, *Z*δ represents facility-level variations, λ denotes the regional fixed effects, τ denotes the year fixed effects, and ϵ is the error term.

A generalized linear model was used to estimate the outcome variables. All outcome variables were treated as continuous such that the estimated coefficients were directly interpreted as the marginal effects. To capture the heterogeneous associations and to provide insights on the potential mechanisms through which insurance-associated differences happened, models were also estimated without controlling for facility type, alternative payment sources, or both.^29^

Sensitivity analysis was performed by excluding the patient records with program interruption or discharge against medical advice and including those records that indicated Medicare as a secondary payer (<1% of the sample). Analysis was also stratified by major subgroups of population (eg, race/ethnicity, early or later aging, low income, and facility type). The seemingly unrelated estimation method was used to compare the estimated differences (TM vs MA) between the stratified groups. SAS statistical software version 9.4 (SAS Institute) was used to conduct data analysis. Data were analyzed from September 2018 to August 2019.

## Results

### Patient Demographic Characteristics

Table 1 provides the summary statistics of the study sample by 2 insurance types, TM and MA, for the 3 treatment conditions. The final sample was 1 028 470 patients (634 619 women [61.7%]; mean [SD] age, 78.23 [7.26] years), including 473 017 patients with stroke, 323 029 patients with hip fracture, and 232 424 patients admitted for joint replacement. Individuals enrolled in MA plans were younger (mean [SD] age, 76.96 [7.02] vs 77.95 [7.26] years for stroke, 79.92 [6.93] vs 80.85 [6.87] years for hip fracture, and 74.79 [6.58] vs 75.88 [6.80] years for joint replacement) and more likely to be black (17 086 [25.5%] vs 54 648 [17.9%] beneficiaries) or Hispanic (14 496 [28.5%] vs 24 377 [8.3%] beneficiaries) than TM beneficiaries; among MA beneficiaries, 20.4% of patients admitted for stroke, 14.1% of those admitted for hip fracture, and 19.5% of those admitted for joint replacement were Hispanic or black. Other patient demographic characteristics and facility characteristics were comparable. For example, for stroke, 28.7% of MA beneficiaries (29 581 patients) and 27.5% of TM beneficiaries (101 913 patients) had comorbidities, 52.0% of MA beneficiaries (53 640 patients) and 52.7% of TM beneficiaries (194 993 patients) were female, and 52.4% of MA beneficiaries (54 117 patients) and 50.1% of TM beneficiaries (185 232 patients) were married. Facilities treating MA and TM beneficiaries had a mean (SD) of 46.04 (34.39) and 46.66 (36.76) beds, respectively, and 21.4% (22 121 facilities) and 27.4% (101 416 facilities), respectively, were freestanding facilities. These variables and differences between TM and MA were fully controlled in the analysis.

**Table 1.** Demographic Characteristics of the Study Sample

Characteristic	Patients, No. (%)					
	Stroke		Hip fracture		Joint replacement	
	MA (n = 103 204)	TM (n = 369 813)	MA (n = 37 160)	TM (n = 285 869)	MA (n = 27 314)	TM (n = 205 110)
LOS, mean (SD), d	14.84 (6.46)	15.13 (6.52)	13.01 (4.79)	13.44 (4.49)	9.68 (3.89)	9.63 (3.61)
FIM score gain, mean (SD)	26.65 (14.86)	26.50 (15.18)	29.41 (13.82)	29.87 (14.26)	31.82 (12.40)	31.81 (12.81)
LOS efficiency, mean (SD), d	2.12 (1.53)	2.08 (1.54)	2.53 (1.43)	2.45 (1.38)	3.72 (1.78)	3.68 (1.76)
Return to community	74 330 (72.0)	249 685 (67.5)	29 310 (78.9)	203 479 (71.2)	25 501 (93.4)	186 915 (91.1)
FIM score at admission, mean (SD)	54.33 (18.22)	54.07 (18.49)	59.86 (14.77)	58.28 (15.10)	70.05 (12.64)	70.28 (13.23)
Comorbidity tier						
None	73 623 (71.3)	267 900 (72.4)	27 285 (73.4)	211 853 (74.1)	20 323 (74.4)	154 964 (75.6)
Minor	26 459 (25.6)	89 635 (24.2)	7122 (19.2)	52 732 (18.5)	6298 (23.1)	45 030 (22.0)
Moderate	1609 (1.6)	6399 (1.7)	2200 (5.9)	16 631 (5.8)	598 (2.2)	4275 (2.1)
Major	1513 (1.5)	5879 (1.6)	553 (1.5)	4653 (1.6)	95 (0.4)	841 (0.4)
Age, mean (SD), y	76.96 (7.02)	77.95 (7.26)	79.92 (6.93)	80.85 (6.87)	74.79 (6.58)	75.88 (6.80)
Female	53 640 (52.0)	194 993 (52.7)	26 297 (70.8)	204 826 (71.7)	17 732 (64.9)	137 131 (66.9)
Hispanic	7807 (7.6)	12 039 (3.3)	3734 (10.1)	7691 (2.7)	2955 (10.8)	4647 (2.3)
Black	13 230 (12.8)	35 117 (9.5)	1477 (4.0)	7552 (2.6)	2379 (8.7)	11 979 (5.8)
Marital status						
Married	54 117 (52.4)	185 232 (50.1)	16 217 (43.6)	119 489 (41.8)	14 638 (53.6)	107 597 (52.5)
Never married	8674 (8.4)	27 547 (7.5)	2713 (7.3)	18 097 (6.3)	2064 (7.6)	13 504 (6.6)
Widowed	38 500 (37.3)	151 243 (40.9)	17 732 (47.7)	144 442 (50.5)	10 249 (37.5)	81 203 (39.6)
Missing	1913 (1.9)	5791 (1.6)	498 (1.3)	3841 (1.3)	363 (1.3)	2806 (1.4)
Alternative payment source	44 701 (43.3)	318 627 (86.2)	15 267 (41.1)	258 273 (90.4)	11 172 (40.9)	193 741 (94.5)
Certified beds, mean (SD), No.	46.04 (34.39)	46.66 (36.76)	43.83 (31.78)	47.02 (36.49)	49.25 (35.48)	50.22 (36.54)
Facility type						
Freestanding	22 121 (21.4)	101 416 (27.4)	10 886 (29.3)	90 433 (31.6)	9757 (35.7)	73 033 (35.6)
In-unit	50 054 (48.5)	176 807 (47.8)	19 070 (51.3)	136 172 (47.6)	13 547 (49.6)	101 660 (49.6)
Missing	31 029 (30.1)	91 590 (24.8)	7204 (19.4)	59 264 (20.7)	4010 (14.7)	30 417 (14.8)

Abbreviations: FIM, Functional Independence Measure; LOS, length of stay; MA, Medicare Advantage; TM, traditional Medicare.

The MA beneficiaries accounted for 21.8% (103 204 of 473 017) of admissions for stroke, 11.5% (37 160 of 323 029) of admissions for hip fracture, and 11.8% (27 314 of 232 424) of admissions for joint replacement. In contrast, according to the Centers for Medicare & Medicaid Services, approximately 31% of beneficiaries were enrolled in MA plans in 2015.^4,5^ eFigure 1 and eFigure 2 in the Supplement show the dynamic changes of total number of patients treated in IRFs and the percentages of MA beneficiaries over the years. Stroke experienced a steady increase in both total number of treatments and percentages of MA beneficiaries. Hip fracture and joint replacement experienced declining total treatment volumes but stable percentages of MA beneficiaries.

### LOS, FIM Score Improvement, and Return to Community

Table 2 and Table 3 show the regressions of major outcome variables on insurance type and control variables for stroke and hip fracture. Results for joint replacement are shown in eTable 1 in the Supplement. Each column in the table represents 1 regression. Coefficients and SEs are reported. Patient demographic and clinical characteristics, facility features, region, and year fixed effects were fully controlled.

**Table 2.** Regression of Care Outcomes on Insurance Type and Control Variables Among Patients Admitted for Stroke

Variable	Model estimated coefficients, mean (SE)				
	FIM score at admission	LOS	FIM score gain	LOS efficiency	Return to community
Medicare Advantage (treatment group)^a^	0.19 (0.04)^b^	−0.17 (0.02)^b^	−0.01 (0.06)	0.02 (0.005)^b^	0.03 (0.002)^b^
FIM score at admission	NA	0.01 (0.001)^b^	0.08 (0.002)^b^	0.01 (0.0002)^b^	0.01 (0.0001)^b^
Comorbidity tier					
None					
Minor	−0.13 (0.03)^b^	−0.02 (0.02)	−1.34 (0.05)^b^	−0.12 (0.005)^b^	−0.03 (0.001)^b^
Moderate	−2.11 (0.10)^b^	1.13 (0.06)^b^	−3.15 (0.16)^b^	−0.33 (0.02)^b^	−0.05 (0.005)^b^
Major	−1.22 (0.11)^b^	−0.01 (0.06)	−3.98 (0.17)^b^	−0.32 (0.02)^b^	−0.06 (0.005)^b^
Age, y	−0.11 (0.002)^b^	−0.02 (0.001)^b^	−0.20 (0.003)^b^	−0.02 (0.0003)^b^	−0.002 (0.0001)^b^
Sex (female, treatment group)^a^	0.48 (0.03)^b^	−0.06 (0.02)^b^	0.001 (0.04)	0.01 (0.004)^c^	0.02 (0.001)^b^
Race/ethnicity					
Hispanic (treatment group)^a^	−1.26 (0.07)^b^	−0.60 (0.04)^b^	−0.84 (0.11)^b^	−0.02 (0.01)^c^	0.10 (0.003)^b^
Black (treatment group)^a^	−0.84 (0.04)^b^	−0.09 (0.03)^b^	−2.01 (0.07)^b^	−0.15 (0.01)^b^	0.04 (0.002)^b^
Marital status					
Married					
Never married	0.02 (0.05)	0.36 (0.03)^b^	0.21 (0.08)^c^	−0.07 (0.01)^b^	−0.12 (0.002)^b^
Widowed	0.18 (0.03)^b^	0.26 (0.02)^b^	0.11 (0.05)^c^	−0.06 (0.005)^b^	−0.08 (0.001)^b^
Missing	−0.02 (0.11)	0.06 (0.06)	−0.03 (0.17)	−0.04 (0.02)^b^	−0.07 (0.005)^b^
Alternative payment source (treatment group)^a^	0.56 (0.04)^b^	0.02 (0.02)	0.05 (0.06)	0.001 (0.005)	−0.01 (0.002)^b^
Certified beds, No.	−0.03 (0.0004)^b^	0.01 (0.0003)^b^	0.01 (0.001)^b^	0.0002 (0.0001)^b^	−0.00004 (0.00002)^c^
Facility type					
Freestanding	−1.94 (0.04)^b^	0.18 (0.02)^b^	5.55 (0.06)^b^	0.42 (0.01)^b^	0.07 (0.002)^b^
Missing	−0.61 (0.10)^b^	−0.11 (0.06)^d^	1.75 (0.15)^b^	0.16 (0.01)^b^	0.03 (0.004)^b^
In-unit					
Observations, No.	473 017	473 017	473 017	473 017	473 017
Control					
Case-mix group	Yes	Yes	Yes	Yes	Yes
Region (region 01 default)	Yes	Yes	Yes	Yes	Yes
Year (2007 default)	Yes	Yes	Yes	Yes	Yes

Abbreviations: FIM, Functional Independence Measure; LOS, length of stay; NA, not applicable.

^a^ Default groups are traditional Medicare; case-mix groups 110, 704, and 802; comorbidity tier none; male; non-Hispanic and nonblack; married; and in-hospital facilities.

^b^ Statistically significant at α = .01.

^c^ Statistically significant at α = .05.

^d^ Statistically significant at α = .10.

**Table 3.** Regression of Care Outcomes on Insurance Type and Control Variables Among Patients Admitted for Hip Fracture

Variable	Model estimated coefficients, mean (SE)				
	FIM score at admission	LOS	FIM score gain	LOS efficiency	Return to community
Medicare Advantage (treatment group)^a^	0.99 (0.06)^b^	−0.11 (0.02)^b^	0.16 (0.08)^c^	0.06 (0.01)^b^	0.05 (0.003)^b^
FIM score at admission	NA	−0.02 (0.001)^b^	−0.05 (0.002)^b^	0.001 (0.0002)^b^	0.01 (0.0001)^b^
Comorbidity tier					
None					
Minor	−0.84 (0.04)^b^	0.70 (0.02)^b^	−1.73 (0.06)^b^	−0.28 (0.01)^b^	−0.05 (0.002)^b^
Moderate	−4.98 (0.07)^b^	1.01 (0.03)^b^	−4.33 (0.10)^b^	−0.49 (0.01)^b^	−0.06 (0.003)^b^
Major	−1.15 (0.14)^b^	1.45 (0.06)^b^	−5.68 (0.19)^b^	−0.72 (0.02)^b^	−0.12 (0.01)^b^
Age, y	−0.26 (0.003)^b^	0.04 (0.001)^b^	−0.25 (0.004)^b^	−0.03 (0.0004)^b^	−0.01 (0.0001)^b^
Sex (treatment group, female)^a^	1.29 (0.04)^b^	−0.15 (0.02)^b^	1.77 (0.05)^b^	0.16 (0.005)^b^	0.03 (0.002)^b^
Race/ethnicity					
Hispanic (treatment group)^a^	−1.72 (0.09)^b^	−0.50 (0.04)^b^	−1.41 (0.13)^b^	−0.07 (0.01)^b^	0.11 (0.004)^b^
Black (treatment group)^a^	−1.62 (0.10)^b^	0.23 (0.04)^b^	−2.92 (0.14)^b^	−0.27 (0.01)^b^	0.03 (0.005)^b^
Marital status					
Married					
Never married	−0.22 (0.07)^b^	0.43 (0.03)^b^	−0.05 (0.10)	−0.13 (0.01)^b^	−0.11 (0.003)^b^
Widowed	−0.18 (0.04)^b^	0.39 (0.02)^b^	−0.14 (0.05)^c^	−0.12 (0.005)^b^	−0.08 (0.002)^b^
Missing	−0.12 (0.15)	0.08 (0.06)	−0.45 (0.20)^c^	−0.07 (0.02)^b^	−0.08 (0.01)^b^
Alternative payment source (treatment group)^a^	0.95 (0.05)^b^	0.11 (0.02)^b^	0.42 (0.07)^b^	0.01 (0.01)	−0.02 (0.002)^b^
Certified beds, No.	−0.02 (0.001)^b^	0.001 (0.0002)^b^	0.01 (0.001)^b^	0.001 (0.0001)^b^	−0.0001 (0.00003)^b^
Facility type					
Freestanding	−3.90 (0.05)^b^	0.38 (0.02)^b^	5.67 (0.07)^b^	0.38 (0.01)^b^	0.06 (0.002)^b^
Missing	−1.93 (0.13)^b^	0.10 (0.05)^d^	2.32 (0.18)^b^	0.17 (0.02)^b^	0.04 (0.01)^b^
In-unit					
Observations, No.	323 029	323 029	323 029	323 029	323 029
Control					
Case-mix group	Yes	Yes	Yes	Yes	Yes
Region (region 01 default)	Yes	Yes	Yes	Yes	Yes
Year (2007 default)	Yes	Yes	Yes	Yes	Yes

Abbreviations: FIM, Functional Independence Measure; LOS, length of stay; NA, not applicable.

^a^ Default groups are traditional Medicare; case-mix groups 110, 704, and 802; comorbidity tier none; male; non-Hispanic and nonblack; married; and in-hospital facilities.

^b^ Statistically significant at α = .01.

^c^ Statistically significant at α = .05.

^d^ Statistically significant at α = .10.

Our results show that MA beneficiaries had a shorter mean LOS for stroke by 0.17 day (95% CI, −0.21 to −0.13 day) (Table 2) and for hip fracture by 0.11 day (95% CI, −0.15 to −0.07 day) (Table 3), which translates to 1.15% (0.17/14.84) and 0.85% (0.11/13.01) differences, respectively, compared with the mean LOS for the MA sample. The mean FIM score improvement during the inpatient stay was not statistically significantly different between TM and MA beneficiaries for stroke (difference, −0.01 unit) (Table 2) but was higher for hip fracture by 0.16 unit (95% CI, 0.01-0.32 unit) among MA beneficiaries (Table 3), which translated into 0.50% (0.16/31.82) higher functional improvements compared with the mean level.

The likelihood of returning to the community after discharge was higher for MA beneficiaries than TM beneficiaries by 3.0% (95% CI, 2.6%-3.4%) for stroke (Table 2) and 5.0% (95% CI, 4.4%-5.6%) for hip fracture (Table 3). eFigure 3, eFigure 4, and eFigure 5 in the Supplement show these adjusted differences between TM and MA over the years.

### Heterogeneous Differences by Insurance Type

Table 4 lists the estimated differences between TM and MA by models with and without fixed effects of facility type, alternative payment sources, or both. Comparison of any pair of the 4 numbers under the same column provides insights on the associations of the 2 subpopulation characteristics with service differences and the potential channels through which these differences happened.

**Table 4.** Insurance Difference With or Without Fixed Effects of Facility Type and Alternative Payment Sources Among Patients Admitted for Stroke and Hip Fracture^a^

Adjusted	Model estimated coefficients, mean (SE)				
	FIM score at admission	LOS	FIM score gain	LOS efficiency	Return to community
Stroke					
No facility type or second payer	0.01 (0.03)	−0.19 (0.02)^b^	−0.20 (0.05)^b^	0.01 (0.01)	0.03 (0.001)^b^
No facility type, with second payer	0.28 (0.04)^b^	−0.18 (0.02)^b^	−0.27 (0.06)^b^	−0.001 (0.006)	0.02 (0.002)^b^
With facility type, no second payer	−0.05 (0.03)	−0.18 (0.02)^b^	−0.03 (0.05)	0.02 (0.005)^b^	0.03 (0.001)^b^
With facility type and second payer	0.19 (0.04)^b^	−0.17 (0.02)^b^	−0.01 (0.06)	0.02 (0.005)^b^	0.03 (0.002)^b^
Hip fracture					
No facility type or second payer	0.59 (0.05)^b^	−0.16 (0.02)^b^	−0.10 (0.07)	0.05 (0.01)^b^	0.05 (0.002)^b^
No facility type, with second payer	1.13 (0.06)^b^	−0.12 (0.02)^b^	−0.004 (0.08)	0.05 (0.01)^b^	0.05 (0.003)^b^
With facility type, no second payer	0.53 (0.05)^b^	−0.16 (0.02)^b^	−0.04 (0.07)	0.06 (0.01)^b^	0.05 (0.002)^b^
With facility type and second payer	0.99 (0.06)^b^	−0.11 (0.02)^b^	0.16 (0.08)^c^	0.06 (0.01)^b^	0.05 (0.003)^b^

Abbreviations: FIM, Functional Independence Measure; LOS, length of stay.

^a^ Entries are model estimated coefficients on insurance type (Medicare Advantage = 1) with SE in parenthesis. Models are adjusted for patient demographic characteristics, clinical conditions, facility characteristics, region, and year fixed effects. Default groups are traditional Medicare; case-mix groups 110, 704, and 802; comorbidity tier none; male; non-Hispanic and nonblack; married; and in-hospital facilities.

^b^ Statistically significant at α = .01.

^c^ Statistically significant at α = .05.

For LOS, fixed effects of facility type and alternative payment sources only minimally absorbed (ie, decreased) the differences between TM and MA, by 0.01 (change in mean [SE] estimated coefficients from −0.19 [0.02] to −0.18 [0.02]) for stroke. For hip fracture, including fixed effects of alternative payment sources decreased the LOS difference between TM and MA by 25% (change in mean [SE] estimated coefficients from −0.16 [0.02] without control to −0.12 [0.02] with control). Including the facility type further decreased the differences. For FIM score improvements, including the fixed effects of facility type in the model almost fully absorbed the differences between TM and MA for stroke (change in mean [SE] estimated coefficients from −0.20 [0.05] to −0.01 [0.06]); in contrast, for hip fracture, including the fixed effects made the insurance differences more salient (change in mean [SE] estimated coefficients from −0.10 [0.07] to 0.16 [0.08]) (Table 4). Differences in the possibility of returning to the community between TM and MA were not substantially changed by including or excluding these 2 fixed effects (change in mean [SE] estimated coefficients, from 0.03 [0.001] to 0.03 [0.002] for stroke and from 0.05 [0.002] to 0.05 [0.003] for hip fracture). Results for joint replacement can be found in eTable 2 in the Supplement.

## Discussion

MA beneficiaries admitted to IRFs had shorter LOSs than did TM beneficiaries without compromising their functional improvements. Facility type and alternative payment sources were shown to partially explain the differences in rehabilitation treatment and care outcomes.

Results from this study correspond with the literature on the association of managed care with rehabilitation health services. First, this study provides a comparison of MA penetration in the rehabilitation field relative to general health care. During the observational period, the percentage of Medicare beneficiaries who enrolled in MA plans increased from 16% to 31%.^4,5^ The sample in this study showed a dynamic pattern in terms of the percentage of MA beneficiaries receiving treatment in IRFs that is consistent with plan enrollment percentages over the years (eFigure 1 and eFigure 2 in the Supplement), yet, the absolute percentage shares in each year were almost universally lower than the shares of MA enrollment, suggesting that proportionately fewer MA beneficiaries (vs TM beneficiaries) were receiving inpatient rehabilitation services. If plan selection issues (either adverse or advantageous) are limited and patient pools are becoming more and more comparable between TM and MA beneficaires,^9^ the differences in percentage shares between the IRF sample and Medicare plan enrollment imply that access to inpatient PAC is still more stringent for MA plans than for TM.

Second, studies^7^ have shown that there are differences in discharge patterns from acute to alternative PAC facilities (eg, home care, SNFs, and IRFs) between TM and MA beneficiaries and differences in the resulting health outcomes on hospital readmission and mortality rates, but results for more direct clinical outcomes such as functional status and recovery are still scarce. This study instead showed that conditional on IRF admission and after controlling for patient and facility characteristics, MA beneficiaries did not experience lower functional improvements than TM beneficiaries did.

Third, although there were differences in LOS and functional improvements between TM and MA beneficiaries, the absolute values were small. The implications are still practically meaningful. On the one hand, a 1.15% shorter LOS for stroke and 0.85% shorter LOS for hip fracture were associated with 3.0% and 5.0% increases, respectively, in the likelihood of returning to the community. On the other hand, because of the large population base of Medicare and the high costs of daily inpatient stay in the US, a 1.15% or 0.85% difference per patient in LOS can yield huge cost savings in total.

Finally, this study provides insights on the potential reasons to explain the differences in care and outcomes between TM and MA beneficiaries.^14,15^ The study showed that shorter LOS and better functional improvements among MA beneficiaries were more likely to happen in freestanding IRFs than in those within the same acute care hospitals, which implies the potential power of care coordination across facilities within managed care networks. These findings were also in line with those of previous studies^13,22^ claiming that facility attributes contributed substantially to care delivery and outcomes. Furthermore, the study shows that shorter LOS and lower functional improvements among MA beneficiaries can be partially explained by the existence of alternative payment sources, implying that the additional payment sources mitigated the differences in care outcomes by Medicare insurance type.

### Limitations

This study has limitations. First, the study sample only included Medicare beneficiaries who were admitted to IRFs for PAC after their hospital inpatient stay. As a result, the differences in care delivery and outcomes between TM and MA enrollees were limited to IRFs. As other studies^7,10^ have shown, differences in PAC between the 2 insurance types are also reflected in discharge patterns to alternative facilities beyond IRFs, such as SNFs and home health agencies. Second, only facility and regional attributes that were available in the UDSMR without restrictions were controlled in the analysis (eg, bed size, facility type, and Centers for Medicare & Medicaid Services region). Including more comprehensive and better refined facility and regional attributes will provide additional insights into the relative strength of patient, facility, and regional factors in driving the differences in care between TM and MA. Third, patient characteristics in the study were limited to basic demographic characteristics and clinical diagnosis. The inclusion of additional patient-level information, such as family history, caregiving structure, and patient perceptions, will help to further explore the channels and mechanisms that contribute to the differences in care between TM and MA.

## Conclusions

This study investigated the differences in inpatient rehabilitation services between TM and MA beneficiaries. The results show that proportionately fewer MA beneficiaries than TM beneficiaries received PAC in IRFs. The MA plan holders experienced shorter LOS, but not worse functional improvements. The possibility of returning to the community was also higher for MA beneficiaries. Freestanding facility type and alternative payment sources were found to be associated with these differences between insurance types.

## Notes

Supplement. **eFigure 1.** Total Number of Patients in IRF by Discharge Year: Stroke, Hip Fracture and Joint Replacement **eFigure 2. **Percentage of Medicare Advantage Patients by Year: Stroke, Hip Fracture and Joint Replacement **eFigure 3.** Adjusted Difference of Length of Stay between TM and MA: Stroke, Hip Fracture and Joint Replacement (Left to Right) **eFigure 4.** Adjusted Difference of FIM Score Improvement between TM and MA7 **eFigure 5.** Adjusted Difference of Likelihood to Return to Community After Discharge Between TM and MA **eTable 1.** Regression of Care Outcomes on Insurance Type and Control Variables: Joint Replacement **eTable 2.** Insurance Difference With/Without Fixed Effects of Facility Type and Alternative Payment Sources: Elective Conditions
