# Supplementary material for: Assessment of Differences in Inpatient Rehabilitation Services for Length of Stay and Health Outcomes Between US Medicare Advantage and Traditional Medicare Beneficiaries
Source: JAMA Netw Open. 2020 Mar 18;3(3):e201204. doi: 10.1001/jamanetworkopen.2020.1204 (PMC7081121; doi:10.1001/jamanetworkopen.2020.1204)
Supplement: Supplement. — eFigure 1. Total Number of Patients in IRF by Discharge Year: Stroke, Hip Fracture and Joint Replacement eFigure 2. Percentage of Medicare Advantage Patients by Year: Stroke, Hip Fracture and Joint Replacement eFigure 3. Adjusted Difference of Length of Stay between TM and MA: Stroke, Hip Fracture and Joint Replacement (Left to Right) eFigure 4. Adjusted Difference of FIM Score Improvement between TM and MA7 eFigure 5. Adjusted Difference of Likelihood to Return to Community After Discharge Between TM and MA eTable 1. Regression of Care Outcomes on Insurance Type and Control Variables: Joint Replacement eTable 2. Insurance Difference With/Without Fixed Effects of Facility Type and Alternative Payment Sources: Elective Conditions [file jamanetwopen-3-e201204-s001.pdf]

Supplementary Online Content

Cao Y, Nie J, Sisto SA, Niewczyk P, Noyes K. Assessment of differences in inpatient rehabilitation services for length of stay and health outcomes between US Medicare Advantage and traditional Medicare beneficiaries. *JAMA Netw Open*. 2020;3(3):e201204. doi:10.1001/jamanetworkopen.2020.1204

eFigure 1. Total Number of Patients in IRF by Discharge Year: Stroke, Hip Fracture and Joint Replacement
eFigure 2. Percentage of Medicare Advantage Patients by Year: Stroke, Hip Fracture and Joint Replacement
eFigure 3. Adjusted Difference of Length of Stay between TM and MA: Stroke, Hip Fracture and Joint Replacement (Left to Right)
eFigure 4. Adjusted Difference of FIM Score Improvement between TM and MA7
eFigure 5. Adjusted Difference of Likelihood to Return to Community After Discharge Between TM and MA
eTable 1. Regression of Care Outcomes on Insurance Type and Control Variables: Joint Replacement
eTable 2. Insurance Difference With/Without Fixed Effects of Facility Type and Alternative Payment Sources: Elective Conditions

This supplementary material has been provided by the authors to give readers additional information about their work.

© 2020 Cao Y et al. *JAMA Network Open.*

eFigure 1: Total Number of Patients in IRF by Discharge Year: Stroke, Hip Fracture and Joint Replacement

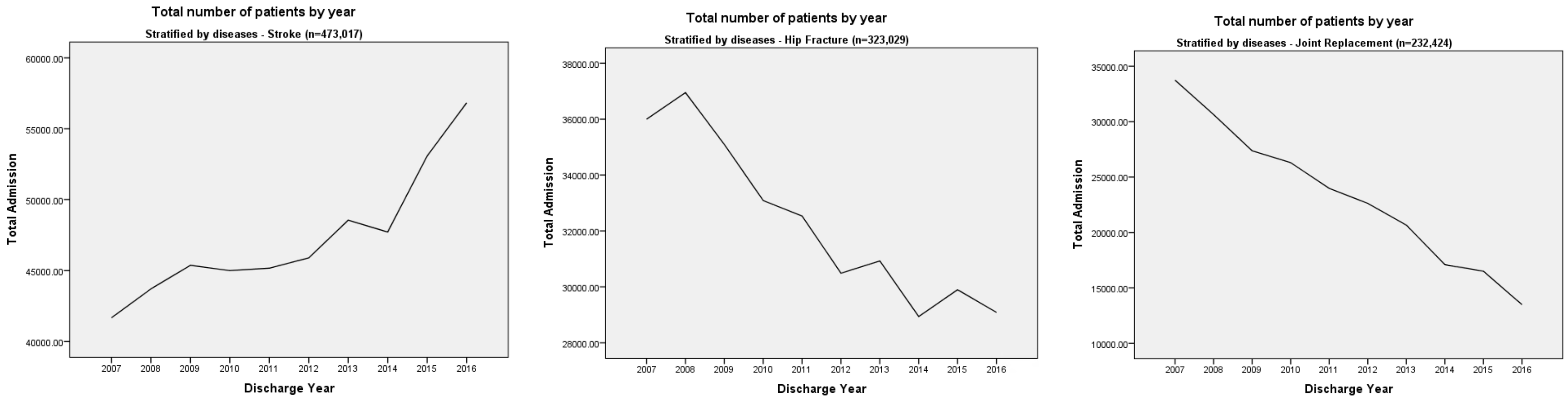

eFigure 2: Percentage of Medicare Advantage Patients by Year: Stroke, Hip Fracture and Joint Replacement

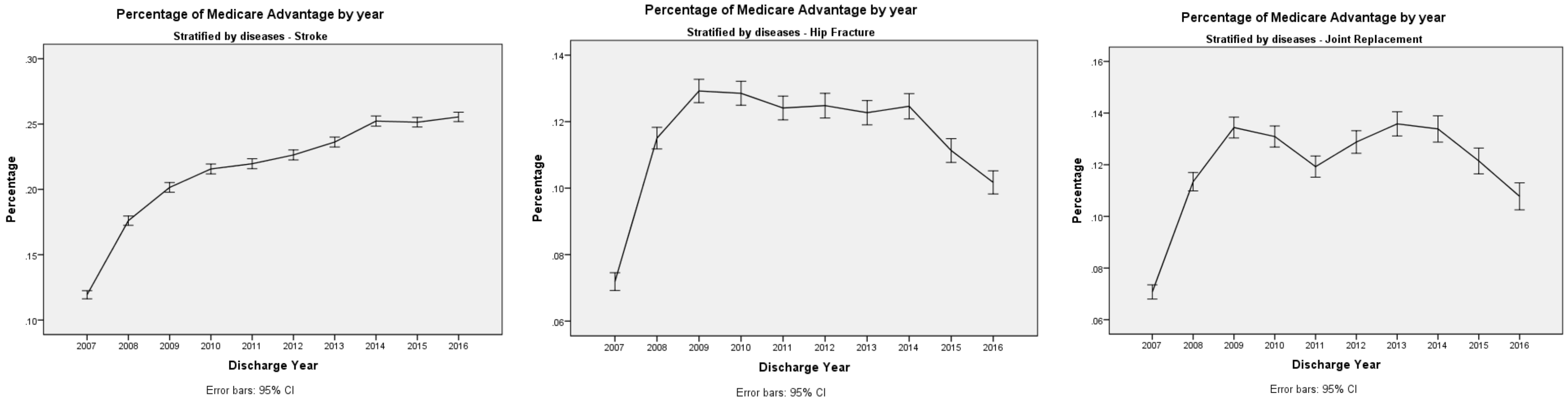

© 2020 Cao Y et al. *JAMA Network Open.*

eFigure 3: Adjusted Difference of Length of Stay between TM and MA: Stroke, Hip Fracture and Joint Replacement (Left to Right)

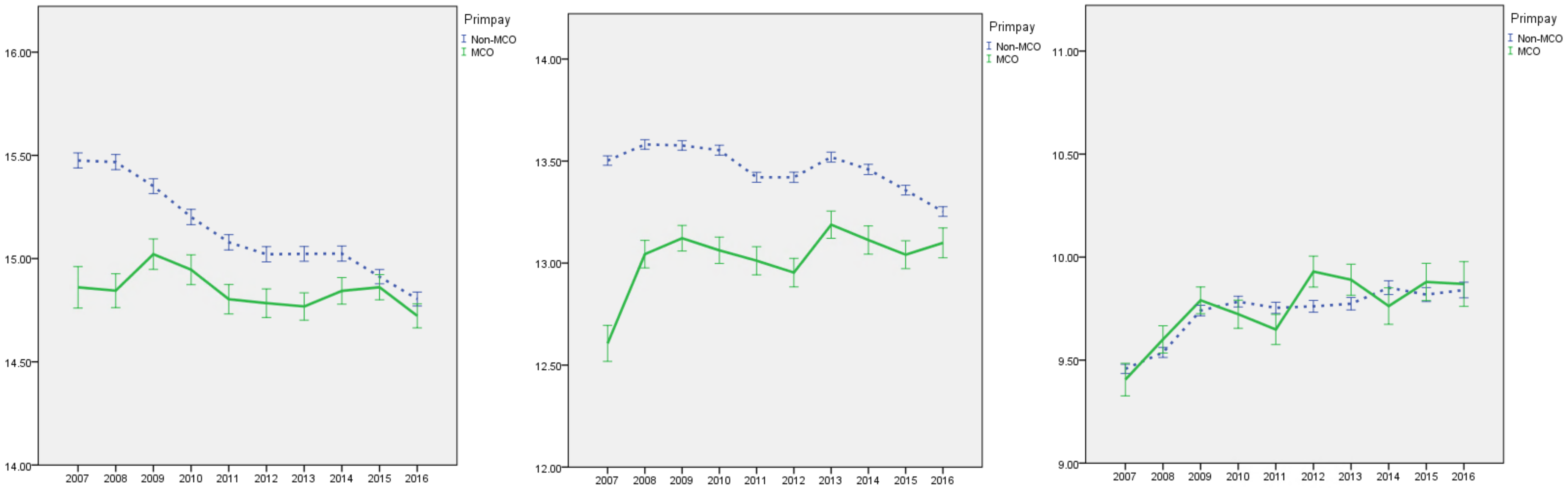

© 2020 Cao Y et al. *JAMA Network Open.*

eFigure 4: Adjusted Difference of FIM Score Improvement between TM and MA

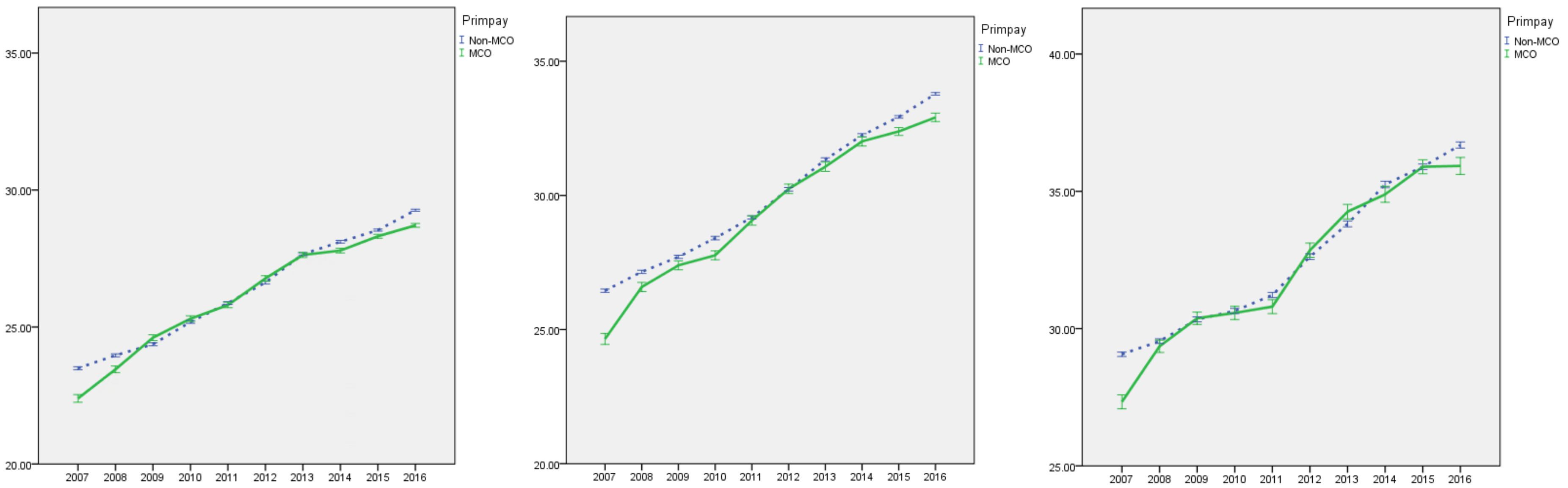

eFigure 5: Adjusted Difference of Likelihood to Return to Community After Discharge Between TM and MA

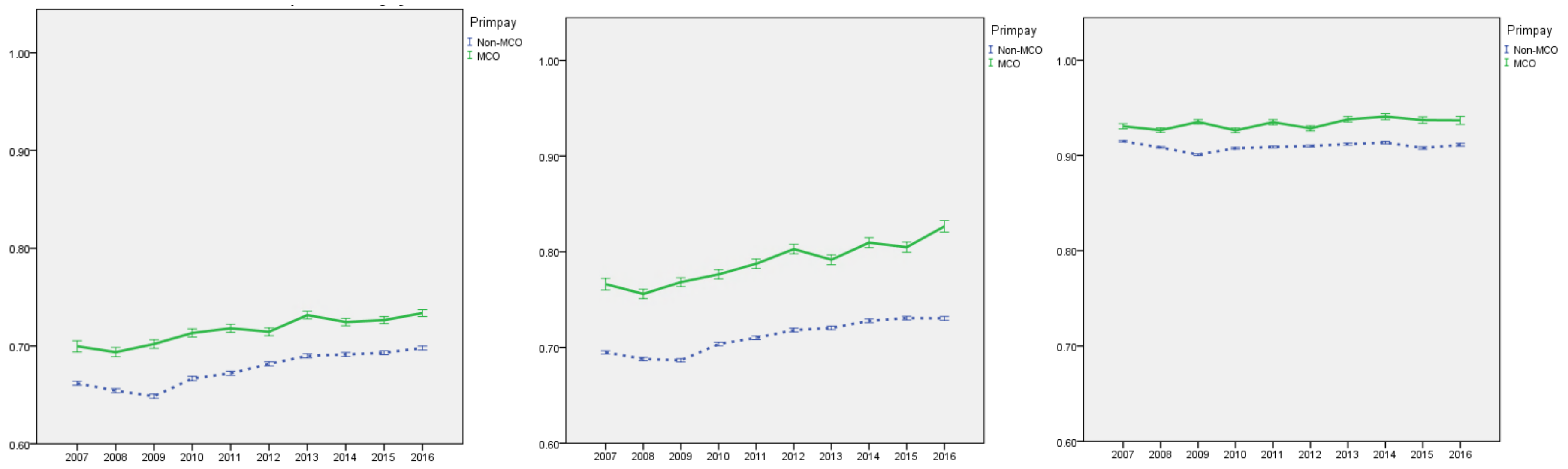

© 2020 Cao Y et al. *JAMA Network Open.*

eTable 1: Regression of Care Outcomes on Insurance Type and Control Variables: Joint Replacement

	FIM Admission	LOS	FIM GAIN	LOS Efficiency	ReturnCommunity
MA(=1)	0.59 (0.05)***	0.05 (0.02)**	-0.06 (0.08)	0.01 (0.01)	0.02 (0.002)***
FIM Admission	NA	-0.04 (0.001)***	-0.48 (0.003)***	-0.05 (0.0005)***	0.003 (0.0001)***
Comorbidity Tier- None					
- Minor	-0.42 (0.04)***	0.63 (0.02)***	-0.55 (0.05)***	-0.30 (0.01)***	-0.02 (0.001)***
- Moderate	-2.08 (0.10)***	1.37 (0.04)***	-3.53 (0.15)***	-0.75 (0.02)***	-0.07 (0.004)***
- Major	-1.64 (0.23)***	1.83 (0.10)***	-5.97 (0.33)***	-1.08 (0.05)***	-0.11 (0.01)***
Age (Years)	-0.20 (0.002)***	0.05 (0.001)***	-0.25 (0.004)***	-0.05 (0.0006)***	-0.003 (0.0001)***
Gender (Female=1)	0.61 (0.03)***	0.11 (0.01)***	0.90 (0.05)***	0.03 (0.01)***	0.01 (0.001)***
Hispanic (=1)	0.04 (0.08)	0.19 (0.04)***	-0.44 (0.12)***	-0.18 (0.02)***	0.01 (0.003)***
Black (=1)	-0.20 (0.06)***	0.62 (0.03)***	-1.04 (0.09)***	-0.33 (0.01)***	-0.01 (0.002)***
Marital Status - Married					
- Never Married	-0.02 (0.06)	0.63 (0.03)***	0.30 (0.09)***	-0.21 (0.01)***	-0.05 (0.002)***
- Widowed	-0.03 (0.03)	0.49 (0.01)***	0.38 (0.05)***	-0.15 (0.01)***	-0.04 (0.001)***
- Missing	0.38 (0.12)***	0.29 (0.05)***	0.14 (0.18)	-0.06 (0.03)**	-0.03 (0.005)***
Alternative Payment Source (=1)	0.59 (0.05)***	0.09 (0.02)***	0.64 (0.07)***	0.01 (0.01)	-0.002 (0.002)
No. of Certified Beds	-0.01 (0.0005)***	0.0002 (0.0002)	-0.004 (0.001)***	-0.00005 (0.0001)	-0.0001 (0.00002)***
Facility type - Freestand	-2.43 (0.04)***	-0.11 (0.02)***	5.02 (0.06)***	0.53 (0.01)***	0.04 (0.002)***
- Missing	-1.09 (0.12)***	-0.15 (0.05)***	1.97 (0.18)***	0.24 (0.03)***	0.01 (0.005)***
- In-Unit					
No. Obs.	232424	232424	232424	232424	232424
Control for …					
CMG	Y	Y	Y	Y	Y
Region (Region01 default)	Y	Y	Y	Y	Y
Year (2007 default)	Y	Y	Y	Y	Y

Entries are model estimated coefficients with standard error in parenthesis
* 90%, ** 95%, *** 99%
Default groups: TM, CMG 110, 704, 802, Tier-None, male, non-Hispanic, non-black, married, in-hospital facilities
TM: Traditional Medicare, MA: Medicare Advantage, LOS: Length of Stay, FIM: Functional Independence Measure

© 2020 Cao Y et al. *JAMA Network Open.*

eTable 2: Insurance Difference With/Without Fixed Effects of Facility Type and Alternative Payment Sources: Elective Conditions

	FIM Admission	LOS	FIM GAIN	LOS Efficiency	ReturnCommunity
Adjusted for …		**Joint Replacement**			
No facility Type or 2nd payer	0.26 (0.05)***	-0.001 (0.02)	-0.37 (0.07)***	0.01 (0.01)	0.02 (0.002)***
No facility Type, with 2nd payer	0.61 (0.05)***	0.05 (0.02)**	-0.10 (0.08)	0.01 (0.01)	0.02 (0.002)***
With facility Type, no 2nd payer	0.27 (0.05)***	-0.001 (0.02)	-0.40 (0.07)***	0.01 (0.01)	0.02 (0.002)***
With facility Type & 2nd payer	0.59 (0.05)***	0.05 (0.02)**	-0.06 (0.08)	0.01 (0.01)	0.02 (0.002)***

Entries are model estimated coefficients on insurance type (MA=1) with standard error in parenthesis
* 90%, ** 95%, *** 99%
Models are adjusted for patient demographics, clinical conditions, facility characteristics, region and year fixed effects
Default groups: TM, CMG 110, 704, 802, Tier-None, male, non-Hispanic, non-black, married, in-hospital facilities
TM: Traditional Medicare, MA: Medicare Advantage, LOS: Length of Stay, FIM: Functional Independence Measure

© 2020 Cao Y et al. *JAMA Network Open.*
